# Effects of Curcumin Analogues DMC and EF24 in Combination with the Cytokine TRAIL against Kidney Cancer

**DOI:** 10.3390/molecules26206302

**Published:** 2021-10-18

**Authors:** Verónica Ibáñez Gaspar, Jasmin McCaul, Hilary Cassidy, Craig Slattery, Tara McMorrow

**Affiliations:** 1 School of Biomolecular and Biomedical Sciences, Conway Institute, University College Dublin, Dublin, Ireland; veronica.ibanezgaspar@ucdconnect.ie (V.I.G.); jasmin.mc-caul@ucdconnect.ie (J.M.); hilary.cassidy@ucd.ie (H.C.); craig.slattery@ucd.ie (C.S.); 2 Systems Biology Ireland, School of Medicine, University College Dublin, Belfield, Dublin, Ireland

**Keywords:** kidney cancer, curcumin, DMC, EF24, TRAIL

## Abstract

The natural compound curcumin has been shown to have therapeutic potential against a wide range of diseases such as cancer. Curcumin reduces cell viability of renal cell carcinoma (RCC) cells when combined with TNF-related apoptosis-inducing ligand (TRAIL), a cytokine that specifically targets cancer cells, by helping overcome TRAIL resistance. However, the therapeutic effects of curcumin are limited by its low bioavailability. Similar compounds to curcumin with higher bioavailability, such as demethoxycurcumin (DMC) and 3,5-bis(2-fluorobenzylidene)-4-piperidone (EF24), can potentially have similar anticancer effects and show a similar synergy with TRAIL, thus reducing RCC viability. This study aims to show the effects of DMC and EF24 in combination with TRAIL at reducing ACHN cell viability and ACHN cell migration. It also shows the changes in death receptor 4 (DR4) expression after treatment with these compounds individually and in combination with TRAIL, which can play a role in their mechanism of action.

## 1. Introduction

Kidney cancer was responsible for 430,000 new cases and almost 180,000 deaths worldwide in 2020 [1]. RCC, the primary form of kidney cancer, is often asymptomatic [2], which together with its complicated clinical manifestation [3], results in high metastasis rates and bad prognosis, with <5% overall 5-year survival [4]. The main therapy for localized RCC is total nephrectomy; however up, to 40% of patients experience metastatic reoccurrence and 5% local reoccurrence [5,6]. Post-operative adjuvant therapies have failed to reduce recurrence in clinical trials and are associated with high toxicity [7,8]. RCC is resistant to traditional chemotherapeutics [3], and the use of targeted therapies is also limited due to multiple drug resistant phenotypes of RCC, highlighting the urgent need for new therapies [9].

TRAIL is a cytokine from the tumor necrosis factor (TNF) superfamily that selectively induces apoptosis in cancer cells without displaying toxicity towards healthy cells [10,11,12,13]. TRAIL binds to membrane receptors DR4 and DR5, activating the extrinsic apoptotic pathway. TRAIL can also activate the intrinsic apoptotic pathway through caspase 8, thus amplifying the apoptotic signal [14]. Although TRAIL presents potential as an immunotherapy [15], many primary cancers are resistant to TRAIL-induced apoptosis [16,17,18]. Mechanisms of TRAIL resistance are unclear but current hypotheses suggest dysregulation of pro- and anti-apoptotic balance, decrease in caspase activity, and defective death receptor signaling or dysregulation of the transcription factor Six1 [19]. Approaches to overcome TRAIL resistance demonstrated high risk of toxicity in vivo [20].

Curcumin ((1E,6E)-1,7-bis (4-hydroxy-3-methoxyphenyl)-1,6-heptadiene-3,5-dione), the primary bioactive extract from *Curcuma longa* (Turmeric), has demonstrated anti-inflammatory, antioxidant, immunomodulatory, anti-tumor and renoprotective activities [21], while being minimally toxic to healthy cells [22,23]. It can sensitize resistant cancer cells to TRAIL through upregulation of DR4 and DR5, promoting TRAIL-induced apoptosis [24]. Recently curcumin was shown to activate apoptosis in kidney cancer cells through reactive oxygen species (ROS) generation and activation of c-Jun N-terminal Kinase (JNK), extracellular signal-regulated kinases (ERK) and p38 [25]. Despite great potential and proven anti-cancer effects, clinical efficacy of curcumin is limited by its low bioavailability and chemical instability [26].

Another turmeric extract, DMC, displayed similar efficacy at inhibiting proliferation and progression in several tumor cell lines [27] and greater potency at preventing cancer cell invasion [28]. Despite its similarities to curcumin, DMC lacks a methoxy group, resulting in improved stability and activity under physiological conditions, making it an attractive potential anti-carcinogenic therapy [29,30]. The synthetic curcumin analogue EF24, shows better pharmacokinetics and anti-cancer and anti-angiogenesis effects, while maintaining minimal toxicity [31]. EF24 has demonstrated high efficacy at growth inhibition in some cancer cell lines, thought to be caused by upregulating oxidative stress and downregulating the nuclear factor kappa-light-chain-enhancer of activated B cells (NFkB), phosphoinositide 3-kinases (PI3K) and mitogen-activated protein kinases (MAPK) pathways [32,33].

## 2. Results

### 2.1. DMC and EF24 Reduce ACHN Cell Viability after 72 h Treatment

**Figure 1:**
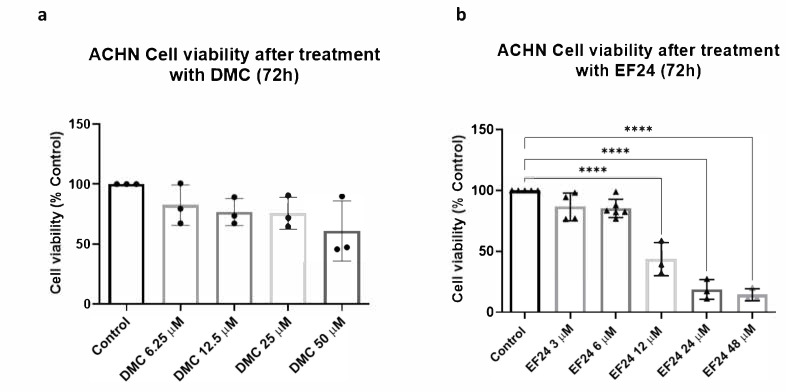
ACHN cell viability measured using MTT assays after 72 h of treatment using either DMC or EF24. (**a**) Cell viability decreases with increasing concentrations of DMC over a 72 h period. Results shown are an average of 3 independent experiments. (**b**) Treatment with increasing concentrations of EF24 reduces ACHN cell viability Cell viability decreases to 43.62% at a 12 µM of EF24 and 18.69% at 24 µM. Results shown are an average of 4 independent experiments. **** *p* ≤ 0.0001.

ACHN cells were treated using increasing concentrations of DMC and EF24. An MTT cell viability assay was performed following 72 h treatment exposure. Increasing concentrations of DMC, between 6.25 and 50 μM, lead to a decrease in ACHN cell viability after 72 h of treatment in a dose-dependent manner (Figure 1a). At the IC_10_ concentration (10.94 µM, previously calculated in a similar experiment using a healthy renal epithelial cell line, see Methods), treatment with DMC decreases cell viability to approximately 76.69% compared to the vehicle control. A similar trend is observed following treatment with increasing concentrations of EF24 (3–48 μM), as ACHN cell viability decreases to approximately 18.69% after treatment with the IC_10_ concentration of EF24 (21.36 µM) (compared to vehicle control) (Figure 1b).

### 2.2. Combination Treatment of DMC or EF24 and TRAIL Further Reduces ACHN Cell Viability

**Figure 2:**
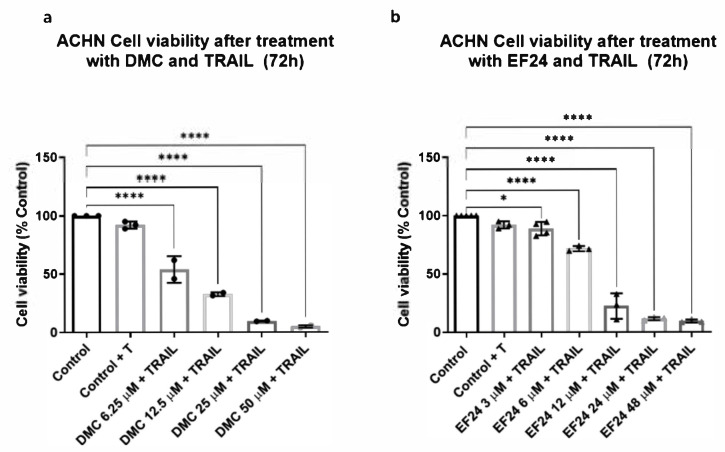
ACHN cell viability measured using MTT assays after 72 h of treatment using either DMC or EF24 in combination with the cytokine TRAIL. (**a**) Cell viability decreases with increasing concentrations of DMC in combination with TRAIL over a 72 h period of time. This decrease is enhanced by addition of TRAIL in comparison to values from Figure 1a. Combination treatment of DMC and TRAIL at 12µM significantly reduces cell viability to 32.73% (**b**) Treatment with increasing concentrations of EF24 in combination with TRAIL reduces ACHN cell viability more than the EF24 monotherapy, up to 22.25% at 12 µM and 11.39% at 24 µM. Results shown are an average of 4 independent experiments. * *p* ≤ 0.05, **** *p* ≤ 0.0001.

ACHN cells were pretreated using DMC or EF24 and a concentration of 50 ng/mL TRAIL was added after 4 h. Cell viability was analyzed after 72 h using an MTT assay. Cell viability of ACHN decreases after combination treatment with DMC and TRAIL in a dose-dependent manner. Cell viability decreases up to 32.73% using a 12 µM concentration of DMC in combination with TRAIL (See Figure 2a). After combination treatment using 24 µM EF24 and TRAIL, cell viability decreases to 11.39% (Figure 2b).

### 2.3. Treatment with DMC or EF24 in Combination with TRAIL Reduces ACHN Cell Migration

**Figure 3:**
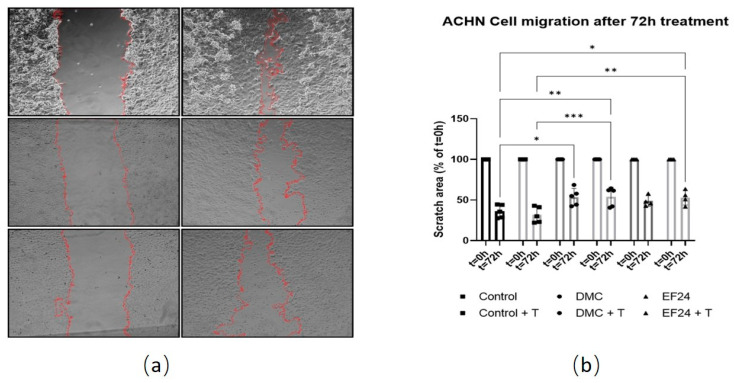
ACHN cell migration measured using a scratch assay at t = 0 h and t = 72 h of treatment using either DMC (10.94 µM) or EF24 (21.36 µM) in combination with TRAIL. (**a**) Scratch area (edges marked in red) at 0 and 72 h after treatment with DMC in combination with TRAIL and EF24 in combination with TRAIL. (**b**) ACHN cell migration decreases in presence of DMC and EF24 both individually and in combination with TRAIL. Combination treatment of DMC and TRAIL reduces scratch width to 54.11% after 72 h, whereas control cells reduce scratch width to 28.78%. Treatment with EF24 in combination with TRAIL reduces scratch width to 53.05%. Results shown are an average of 4 independent experiments. * *p* ≤ 0.05, ** *p* ≤ 0.01, *** *p* ≤ 0.001. Images analyzed using ImageJ [34].

A scratch cell migration assay was performed using ACHN cells treated with either DMC or EF24. Images were taken at 0 and 72 h post-scratch and scratched, cell-free area was measured to see if treatment slowed scratch closure compared to control (Figure 3a). Scratch area is decreased for all samples at 72 h, indicating that the gap is closing. Scratch area is wider in the presence of DMC or EF24 treatment compared to control. After 72h treatment, EF24 (48.67%) and EF24+TRAIL (53.05%) significantly reduces ACHN migration rate compared to control (28.78%). DMC and DMC+TRAIL treatments also reduce the migratory potential compared to control, being the scratch area 50.62% and 54.11% (Figure 3b).

### 2.4. DR4 Expression Increases after 72 h Treatment with EF24 in Combination with TRAIL

**Figure 4:**
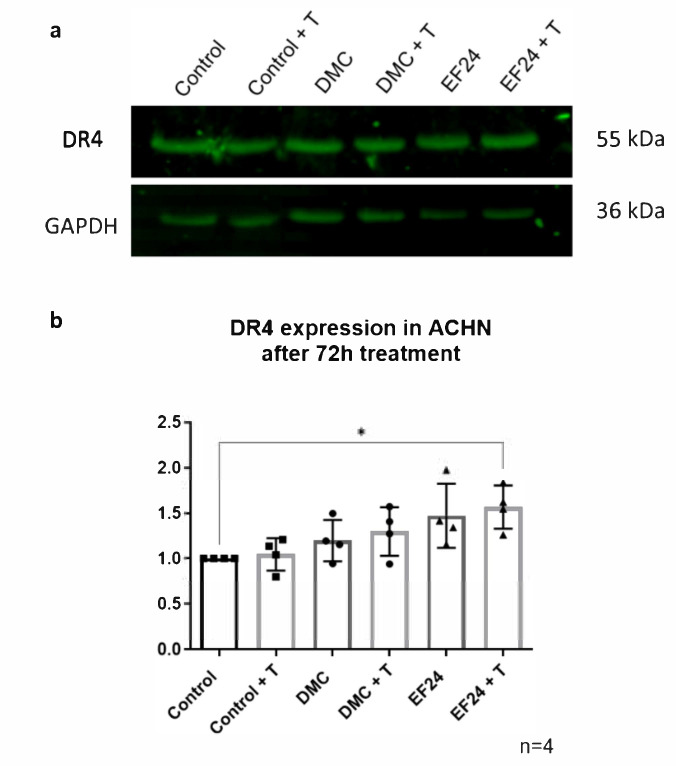
DR4 expression in ACHN after 72 dh treatment with the IC_10_ concentrations of DMC or EF24 either individually or in combination with TRAIL. (**a**) The expression of DR4 is increased after each treatment, especially after combination treatment with EF24 and TRAIL. GAPDH was used as a loading control. Represented results were normalized using the control. (**b**) Representative blots of DR4 expression in ACHN after treatment with DMC or EF24 individually and in combination with TRAIL. Images obtained using Odyssey CLx system. All data shown is n = 3. Densitometry analysis performed using ImageJ [34] and GraphPad Prism. * *p* ≤ 0.05.

Western blot analysis of ACHN cells after 72 h of treatment with the IC_10_ concentrations of DMC or EF24 individually and in combination with TRAIL showed an increase in DR4 protein expression after treatment with Curcumin, DMC, DMC + TRAIL, EF24 and EF24 + TRAIL (Figure 4).

## 3. Discussion

The natural compound curcumin has previously shown multiple anti-cancer effects against several different cancer cell types, such as breast cancer, lung cancer or pancreatic cancer [35,36,37]. Recently, it was demonstrated that curcumin enhances the pro-apoptotic effect of TRAIL thus leading to induction of apoptosis through an upregulation of DR4 and DR5, two TRAIL receptors [24]. This effect was also observed in the RCC cell line ACHN, which takes place through activation of JNK and ERK and ROS generation [25]. However, due to the low bioavailability displayed by curcumin, its therapeutic potential is severely limited.

In this study, we analyzed the anti-cancer potential of the two curcumin analogues DMC and EF24 alone and in combination with TRAIL in a RCC cell line. These two analogues have previously demonstrated a better bioavailability compared to curcumin due to their chemical and structural differences and seem to present anti-cancer activity against several cancer cell lines [27,32,33]. However, the effects of these two compounds have not been studied in RCC cells and thus have their potential to re-sensitize TRAIL-resistant cells to the effects of the cytokine.

Figure 1a shows that the natural curcuminoid DMC led to a reduction in cell viability of the RCC cell line ACHN. This effect was dose dependent, as the reduction of cell viability increased with increasing drug concentration. The IC_10_ concentration of DMC, 10.94 µM, which was previously calculated in a normal renal epithelial cell line (RPTEC/TERT1), led to a reduction of ACHN cell viability to 76.69% compared to the vehicle control. After treatment with the synthetic curcuminoid EF24, ACHN cell viability decreased in a dose-dependent manner as well (Figure 1b). In the case of EF24, the IC_10_ concentration calculated in RPTEC/TERT1, 21.36 µM, led to a reduction of cell viability in RCC cells of 18.69%. These two compounds used individually could have some therapeutic potential against RCC at concentrations that do not significantly damage healthy renal epithelial cells.

Treatment of ACHN RCC cells using curcumin and the cytokine TRAIL enhanced the effect of both compounds individually, reducing ACHN cell viability. In this study, the synergy between TRAIL and the curcuminoids DMC and EF24 was studied following the protocol from *Obaidi* et al. ACHN cells treated with only 50 ng/mL TRAIL did not show a significant decrease in cell viability, meaning this cell line is resistant to TRAIL-induced apoptosis. When ACHN were treated using a combination of TRAIL and DMC or EF24, there was a decrease in ACHN cell viability. The combination treatment using DMC and TRAIL reduced cell viability at all DMC concentrations tested, resulting in viability percentages lower than after treating cells with DMC individually (Figure 2a). At the IC_10_ concentration of DMC, 10.94 µM, the combination treatment of DMC and TRAIL reduced ACHN viability to 32.73%, lower than half of the cell viability percentage after treatment with DMC individually. Similar trends were observed when ACHN cells were treated with a combination of EF24 and TRAIL, as cell viability was lower after combination treatment compared to the treatment with EF24 only. In this case, ACHN cell viability was reduced to 11.39% after treatment with the IC_10_ concentration of EF24 in combination with TRAIL, compared to 18.69% of the individual treatment with EF24 (Figure 2b). These findings point to the potential of DMC and EF24 to re-sensitize resistant cells to TRAIL-induced apoptosis, as the decrease in cell viability is enhanced when TRAIL is combined with either DMC or EF24.

RCC is a highly metastatic cancer, often diagnosed at late stages [3]. *Obaidi* et al. showed that the combination of curcumin and TRAIL can reduce ACHN cell migration in a zebrafish model. The effects of DMC and EF24 individually and in combination with TRAIL were studied regarding their potential to reduce ACHN cell migration in vitro. As seen in Figure 3, the area of a scratch on a ACHN cell monolayer was measured over a period of 72 h. ACHN treated only with TRAIL did not significantly show a difference in scratch area after 72 h compared to the vehicle control ACHN, meaning treatment with only TRAIL does not alter the migration potential of ACHN cells. When using DMC individually and in combination with TRAIL, the scratch area after 72 h treatment comprised 54.11%, almost twice as wide as the control cells (28.78% at 72 h treatment). This is also observed after treatment with EF24 and EF24 in combination with TRAIL, where the scratch area was almost twice (53.05%) the area of the control ACHN after 72 h treatment (Figure 3). This points to the potential of DMC and EF24 treatment to reduce cell migration of ACHN, although in vivo experiments should be performed in order to assess whether this effect also occurs in vivo. TRAIL did not seem to play a role in cell migration reduction in combination with these two compounds in ACHN.

It is known that curcumin presents a wide variety of cellular targets that play a role in key functions such as cell survival and apoptosis [38,39,40]. In ACHN, curcumin in combination with TRAIL was shown to upregulate the expression of the TRAIL receptor DR4, which could explain the decrease in cell viability after combination treatment.

The effects observed after treatment using DMC and EF24 individually and in combination with TRAIL may occur through similar mechanisms due to their analogy to curcumin; however, these compounds are much less studied and could affect cells through different mechanisms. This study analyzed the changes in DR4 expression in ACHN cells after treatment. As seen in Figure 4a, the expression of DR4 increased in ACHN after treatment with EF24 and significantly increased after treatment with EF24 in combination with TRAIL. This suggests EF24 helps overcome TRAIL resistance in ACHN cells by upregulating DR4 expression, which leads to decreased cell viability in this cell type. After treatment with DMC alone and in combination with TRAIL, levels of DR4 expression remained similar to the control ACHN expression levels. This shows that both curcuminoids present a different mechanism of action, which is not necessarily similar to curcumin. Although further analysis is necessary in order to elucidate what cell changes take place after treatment with DMC and EF24, this research shows the potential anticancer effects in RCC of the curcumin analogues DMC and EF24 and how they may be able to help overcome TRAIL resistance in this cell line while reducing cell migration. These changes in DR4 expression are currently the only known possible steps in the mechanism of action of either DMC or EF24 in the ACHN cell line; however, preliminary analysis of protein expression points to changes in the expression of caspase 8 and the pro-apoptotic protein Bax. Further research into these two potential changes may potentially explain the decrease in cell viability observed after treatment with these two compounds.

## 4. Materials and Methods

### 4.1. Cell Culture

A human RCC cell line, ACHN (ATCC, Manassas, VA, USA) was used. ACHN cells were cultured in Minimum Essential Medium (Merck, Darmstadt, Germany) with 1% Penicillin-streptomycin (Thermo Fisher, Dublin, Ireland) and 10% fetal bovine serum (Thermo Fisher, Dublin, Ireland). Cells were stored at 37 °C and 5% CO_2_. Cells were sub-cultured at 80–85% confluency using pre-warmed trypsin (Thermo Fisher, Dublin, Ireland), PBS (Merck, Darmstadt, Germany) and cell medium. For the experiments, 6-well plates were seeded at a density of 2 × 10^5^ cells/well 24-well plates were seeded at a density of 4 × 10^4^ cells/well.

### 4.2. Cell Treatments

For the experiments, cells were pre-treated with either DMC (Merck, Darmstadt, Germany), and EF24 (Merck, Darmstadt, Germany) both in DMSO (Merck, Darmstadt, Germany) and 50 ng/mL of TRAIL (Merck, Darmstadt, Germany) (in PBS) was added to wells after 4 h. The IC_10_ and IC50 concentrations of DMC (10.94 µM and 25.30 µM +/− 1.89 µM SEM) and EF24 (21.36 µM and 30.60 µM +/− 2.39 µM SEM), previously calculated using RPTEC/TERT1 (ATCC). In order to ensure that the treatments are not toxic to a high percentage of healthy kidney cells, the IC_10_ concentrations were chosen to proceed with. A treatment time of 72 h was chosen, as it was the time at which the maximum decrease in cell viability in RPTEC prior to cell doubling time was seen.

### 4.3. MTT Cell Viability Assay

Cells were treated (see Methods above) in duplicate using increasing concentrations of DMC or EF24. Plate was incubated for 72 h at 37 °C, 5% CO_2_. MTT (Thermo Fisher, Dublin, Ireland) solution was diluted using pre-warmed cell media to a working concentration of 0.5 mg/mL and 500 µL of working MTT solution was added to each well after old medium was removed. The plate was incubated in the dark for 2 h at 37 °C. The MTT solution was discarded and 500 µL of DMSO was added to each well and plate was incubated for 1h at room temperature. Absorbance was read at 570 nm using CLARIOstar plate reader (BMG Labtech, Ortenberg, Germany).

### 4.4. Cell Migration Assay

Cells were cultured until a confluent monolayer was formed. Each well was scratched vertically down the center using a p200 pipette tip. Cells were then washed with PBS and treated (see Methods above). Images were taken using an Olympus E50 microscope and scratch width was recorded for time point 0 h measurements. Cells were incubated at 37 °C and images and measurements were recorded at 24, 48 and 72 h.

### 4.5. Western Blot

Cells were cultured and treated in 6-well plates. After 72 h treatment, whole cell lysates were performed and frozen at −20 °C until used. Electrophoresis was performed using 12% gels at 80 V/110 V using the BioRad MiniProtean Electrophoresis system. Wet transfer was performed using a nitrocellulose membrane and the BioRad mini trans-blot cell system on ice at 100 V for 90 min. Membranes were blocked using a 5% Marvel in TBS-T solution and incubated at 4 °C overnight with DR4 (Cell Signaling Technology, Boston, MA, USA) primary antibody diluted in a 5% Marvel in TBS-T solution (1:2000). Membranes were then washed with TBS-T (three times, 10 min each) and incubated with IRDye 800CW goat anti-rabbit IgG (LI-COR Biotechnology, Cambridge, UK) secondary antibody (1:20,000) at room temperature, covered in foil for 1 h. Membranes were washed three times, 5 min each and detected using the Odyssey CLx system (LI-COR Biotechnology, Cambridge, UK).

### 4.6. Statistical Analysis

Migration assay scratch area calculation and densitometry analysis were preformed using ImageJ Fiji [34]. GraphPad Prism 9.0 (GraphPad Software, San Diego, CA, USA) was employed to analyze all data. MTT and western blot data was analyzed using one-way ANOVA and a post hoc Tukey’s multiple comparisons test. Migration assay data was analyzed using two-way ANOVA and a post hoc Tukey’s multiple comparisons test. Results are shown as mean ± SEM. A *p* value of 0.05 or smaller was deemed statistically significant. Statistical significance was represented by * (*p* < 0.05); ** (*p* < 0.01); *** (*p* < 0.001); **** (*p* < 0.0001).

## 5. Conclusions

Curcumin has been studied for its anti-cancer effects, but due to its low bioavailability, its therapeutic use is limited. This study shows that the curcumin analogues with better bioavailability, DMC and EF24, decrease in vitro cell viability and cell migration of RCC cells (ACHN). These effects are further enhanced when in combination with TRAIL, pointing to DMC and EF24 as potential compounds to overcome TRAIL resistance in kidney cancer cells. The mechanisms are yet to be elucidated; however, an increase in TRAIL receptor expression (DR4) may play a role in kidney cancer cell resensitization to TRAIL. Further analysis of protein expression of pro- and anti-apoptotic proteins, such as caspases and proteins from the Bcl-2 family, may potentially help understand the mechanism of action of these compounds in the ACHN cell line.

## Data Availability

Data available upon request from corresponding authors.

## Sample Availability

Not available.
